# Recognition of the novel items for prediction of bone metastasis in colorectal cancer

**DOI:** 10.3389/fonc.2025.1666891

**Published:** 2025-10-21

**Authors:** Min Chen, Xuan Wang, Shuheng Bai, Ning Lan, Junyang Wang, YanKe Chen, Ying Gao, WenJuan Wang, Xiao Shang, Min Jiao, Xiangxiang Zhang, Wenyang Li, Fang Wu, Wanyi Liu, Fengyuan Hu, Ling Chen, Juan Ren

**Affiliations:** ^1^ Department of Radiotherapy Oncology, the First Affiliated Hospital of Xi’an Jiaotong University, Xi’an, China; ^2^ School of Basic Medical Sciences of Xi’an Jiaotong University, Xi’an, China; ^3^ Department of Oncology, the First Affiliated Hospital of Xi’an Jiaotong University, Xi’an, China; ^4^ Department of Medical Oncology, the First Affiliated Hospital of Xi’an Jiaotong University, Xi’an, China

**Keywords:** colorectal cancer, bone metastasis, uric acid, neutrophil/lymphocyte ratio, uric acid/albumin ratio

## Abstract

**Objective:**

To explore whether uric acid (UR), neutrophil/lymphocyte ratio (NLR) and uric acid/albumin ratio (UAR) can predict bone metastasis in colorectal cancer (CRC).

**Methods:**

A single-center retrospective study was conducted studying patients diagnosed with colorectal cancer attending The First Affiliated Hospital of Xian JiaoTong University between January 2016 and December 2021. Patients were categorized into groups with and without bone metastasis. Receiver operating characteristic (ROC) curve analysis assessed the diagnostic accuracy of CRC bone metastases, with subsequent combined ROC curve analysis. Differences among the AUCs were calculated and compared by Delong test. Logistic regression analysis was utilized to assess the impact of these parameters on CRC bone metastasis.

**Results:**

A total of 156 patients (32%) exhibited bone metastases from CRC. In these patients, levels of uric acid (UA), uric acid ratio (UAR), neutrophil-to-lymphocyte ratio (NLR), carcinoembryonic antigen (CEA), carbohydrate antigen 199 (CA199), and carbohydrate antigen 724 (CA724) were significantly elevated. The diagnostic performance of UA, UAR and NLR is surpassed that of traditional colorectal cancer markers. The area under the curve (AUC) for the combination UA, UAR and NLR with colorectal cancer tumor markers was significantly more effective in predicting bone metastasis (*P* < 0.001) compared to the AUC without this combination. Multiple logistic regression analysis identified UA, NLR and CEA as independent risk factors for bone metastasis in colorectal cancer.

**Conclusions:**

UA, UAR and NLR serve as valuable makers for predicting bone metastases in patients with colorectal cancer. The integration of UA, UAR, NLR, CEA, CA199 and CA724 may enhance the prediction of bone metastases in colorectal cancer.

## Introduction

Colorectal cancer (CRC) ranks as the second deadliest cancer globally, with 1.93 million new cases and 903,859 deaths reported in 2022, according to Global Cancer Data statistics (1). Approximately 3-7% of colorectal cancer patients develop bone metastases (2). Nevertheless, routine follow-up does not include screening for bone metastases in colorectal cancer (3). Diagnosis typically occurs through targeted imaging following the emergence of bone-related events, such as pathological fractures, severe bone pain, or spinal cord compression. Once bone metastasis occurs in colorectal cancer patients, the prognosis is dire, with a 5-year survival rate of less than 5% and a median survival time ranging from 5 to 21 months (4). Furthermore, bone metastases associated with bone-related events significantly impair patients’ quality of life and are compounded by a lack of effective interventions and treatments. Consequently, there is an urgent need for timely, effective and non-invasive monitoring of bone metastases occurrence in colorectal cancer patients.

Serum uric acid (UA), the serum uric acid/serum albumin ratio (UAR), and the neutrophil/lymphocyte ratio (NLR) are biochemical markers that are easily measurable, cost-effective, and non-invasive for patients. UA is the final product of purine metabolism, generated through the oxidation of various purines and subsequently excreted in urine. Increasing evidence suggests that elevated UA levels serve as a risk factor for several cancers by inducing inflammatory responses and oxidative stress (5, 6). Albumin, the principal component of serum protein, reflects nutritional status and cancer aggressiveness and is frequently incorporated into prognostic scoring systems in numerous studies (7). Neutrophils, as key components of white blood cells, significantly contribute to cancer progression and have emerged as independent risk factors for various malignant tumors (8, 9), closely associated with tumor metastasis (10). However, the relationships among UA, UAR and NLR, particularly concerning bone metastasis in colorectal cancer have not been systematically investigated. Therefore, this study aimed to utilize retrospective data to examine the diagnostic utility of UA, UAR and NLR in identifying bone metastasis in colorectal cancer patients, facilitating timely and non-invasive detection to enhance patient quality of life and improve survival rates.

## Materials and methods

### Participant selection

This study was a single-center, retrospective analysis of patients with colorectal cancer bone metastasis, diagnosed through pathology and who had not received any form of treatment, including surgery, radiotherapy, chemotherapy, molecular targeted therapy, or immunotherapy, at the First Affiliated Hospital of Xi’an Jiaotong University in Shaanxi, China. We utilized hospital records form the First Affiliated Hospital of Xi’an Jiaotong University to identify all patients diagnosed between January 2016 and December 2021. The ICD-10 diagnostic codes C18–20 were employed to extract patient data from the electronic records. A researcher reviewed these hospital records to gather information on gender, age, serum uric acid levels, bone metastasis status, neutrophil/lymphocyte ratio, albumin levels, and the uric acid/albumin ratio among other variables.

Patients were eligible for this study if they satisfied the following criteria:

1. A diagnosis of colorectal cancer was confirmed through pathological examination.
2. The patient presented for their first visit without any prior treatment, including surgery, radiation therapy, chemotherapy, molecular targeted therapy, or immunotherapy.
3. The medical records were complete, including blood routine and biochemical test reports obtained within three weeks prior to the pathological examination.

Patients were excluded if they met any of the following criteria:

1. A history of gout or other conditions associated with pathologically elevated uric acid levels.
2. The presence of other malignant tumors or platelet-related disorders.
3. Severe hepatic or renal insufficiency.
4. Recent or long-term use of glucocorticoids.

Our study used retrospective data to screen potential patients based on the inclusion and exclusion criteria confirmed by the initial research design. Then, the patient was divided into bone metastasis group and non-bone metastasis group according to whether there was bone metastasis. Finally, a total of 488 patients were included in this study, of which 156 were in the bone metastasis group and 332 were in the non-bone metastasis group. Therefore, we used the non-bone metastasis group as the negative group and the bone metastasis group as the positive group, and a series of subsequent analyses were also based on this grouping.

### Hematology and biochemical index detection

For patients with colorectal cancer bone metastasis who had not received any prior treatment, including anti-tumor therapies such as surgery, radiotherapy, chemotherapy, molecular targeting and immunotherapy, peripheral venous blood was drawn after an 8-hour fasting period. The sample were sent to our hospital’s laboratory for analysis, adhering strictly to the instrument and reagent instructions. The serum uric acid test utilized the JDYFY-SH-YQA-25 instrument and its corresponding reagents. The normal range for serum uric acid in males was 208-428umol/L, while in females, it was 155-357umol/L. The normal values for neutrophil count and lymphocyte count were 1.8-6.3*10^9^/L and 1.1-3.2*10^9^/L, respectively, and the normal range for albumin was 40–55 g/L. Neutrophil count (N) and lymphocyte count (L) were measured using an automated hematology analyzer (BC-6800Plus). The neutrophil/lymphocyte ratio (NLR) was calculated as N/L, and the uric acid/albumin ratio was calculated as UA/Ab.

### Diagnostic criteria for bone metastases of colorectal cancer

According to the expert consensus on the Multidisciplinary Comprehensive Treatment of Colorectal Cancer Bone Metastases in China (2020 edition) (11), the diagnosis of colorectal cancer bone metastases must satisfy one of the following two criteria:

1. A clinical or pathological diagnosis of colorectal cancer, with a bone lesion biopsy confirming colorectal cancer metastasis;
2. A clear pathological diagnosis of colorectal cancer accompanied by typical imaging findings indicative bone metastases.

### Statistical analysis

The Kolmogorov-Smirnov test was conducted on the continuous data prior to analysis to assess the normality of the variables. Continuous variables were presented as mean ± standard deviation (SD), while categorical variables were expressed as percentages. The independent sample t-test was employed for continuous variables exhibiting a normal distribution, whereas the Mann-Whitney rank sum test was utilized for data that did not follow a non-normal distribution. Count data were analyzed using the Chi-square test. Correlation analysis was performed using the Spearman method. Multiple logistic regression analysis was applied to identify factors potentially associated with bone metastasis in colorectal cancer. A receiver operating characteristic (ROC) curve and area under the curve (AUC) value were employed to evaluate the sensitivity and specificity of each factor in assessing colorectal cancer bone metastasis. Differences among the AUCs were calculated and compared using the Delong test. All statistical analyses were performed using SPSS version 26 (IIBM Corporation, Armonk, NY). A *P*-value of less than 0.05 was considered statistically significant.

## Results

**Figure 1:**
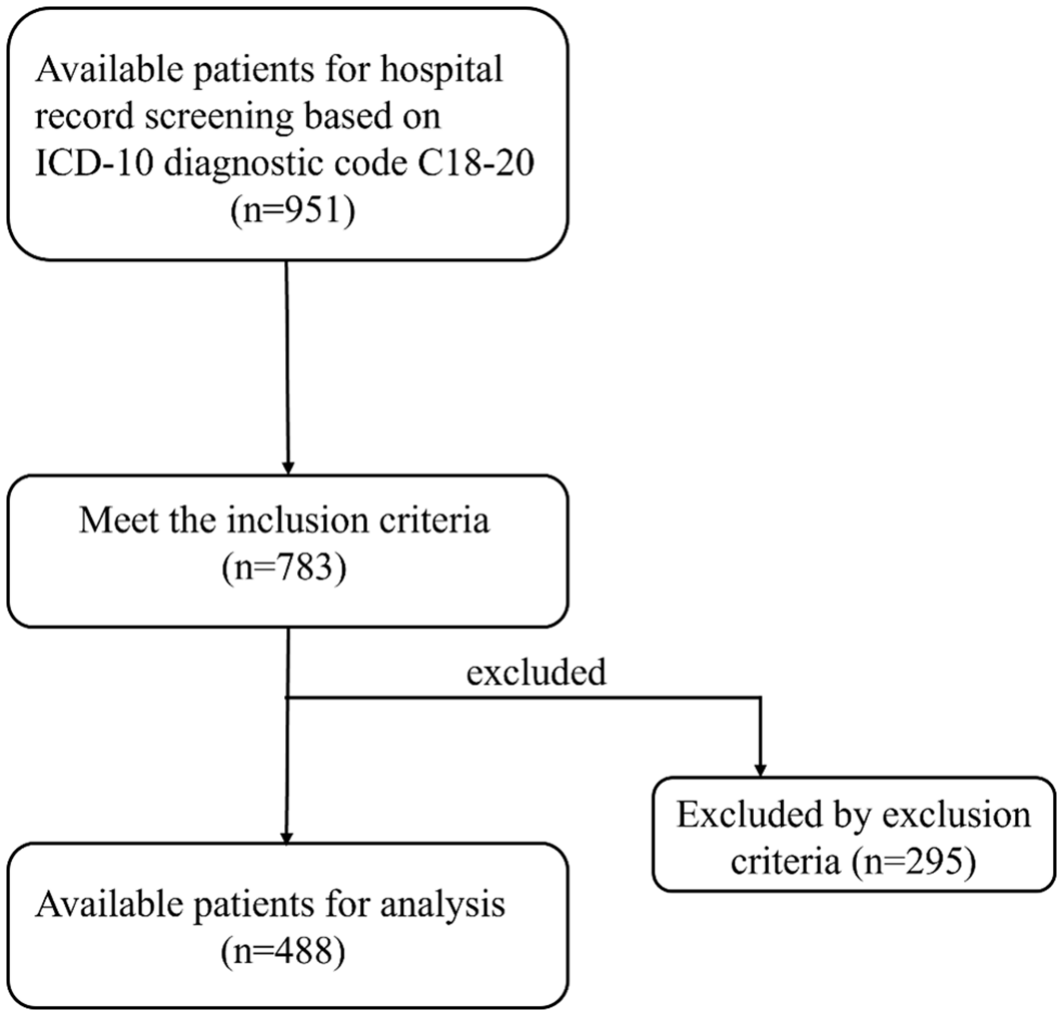
Flow chart of patient selection.

A total of 488 patients who met the inclusion and exclusion criteria were enrolled in this study (**Figure 1**). This cohort comprised 364 males (74.6%) and 124 females (25.4%), with 156 patients (32.0%) exhibiting bone metastasis (metastasis group) and 332 patients (68.0%) without bone metastasis (non-metastasis group). Results from the Kolmogorov-Smirnov test indicated that age, UA, UAR, CEA, CA199, CA724 and NLR exhibited non-normal distributions between the two groups (**Table 1**). Consequently, these data were reported as medians (P25-P75) and compared between groups using the rank sum test. No statistically significant differences were observed in age, gender, albumin, leukocyte and monocyte counts between the two groups. However, levels of CEA, CA199 and CA724 in the bone metastatic group were significantly higher than those in the non-metastatic group (*P*< 0.001, *P* = 0.006, *P* = 0.005). The metastasis group also demonstrated significantly increased absolute UA and decreased absolute albumin, resulting in a significantly elevated UAR (*P*< 0.001). Additionally, the neutrophil count in the metastasis group was elevated, while the lymphocyte count also increased (*P* = 0.004, *P* < 0.001) (**Table ​2**).

**Table 1:** Kolmogorov-Smirnov test of measurement data.

Measurement data	Group	Statistical value	Significance
Age	Non-metastasis	0.059	0.008
	Metastasis	0.089	0.004
Albumin, g/L	Non-metastasis	0.028	0.2
	Metastasis	0.064	0.2
Globulin, g/L	Non-metastasis	0.044	0.2
	Metastasis	0.052	0.2
Uric acid, umol/L	Non-metastasis	0.052	0.032
	Metastasis	0.106	<0.001
UAR	Non-metastasis	0.086	<0.001
	Metastasis	0.103	<0.001
CEA, ng/mL	Non-metastasis	0.435	<0.001
	Metastasis	0.442	<0.001
CA199, U/mL	Non-metastasis	0.303	<0.001
	Metastasis	0.405	<0.001
CA724, U/mL	Non-metastasis	0.379	<0.001
	Metastasis	0.405	<0.001
Lymphocyte, 10^9^/L	Non-metastasis	0.067	0.001
	Metastasis	0.178	<0.001
Neutrophil, 10^9^/L	Non-metastasis	0.166	<0.001
	Metastasis	0.118	<0.001
NLR	Non-metastasis	0.238	<0.001
	Metastasis	0.298	<0.001
Erythrocyte, 10^12^/L	Non-metastasis	0.342	<0.001
	Metastasis	0.09	<0.001
Hemoglobin, g/L	Non-metastasis	0.086	<0.001
	Metastasis	0.153	<0.001
Leukocyte, 10^9^/L	Non-metastasis	0.121	<0.001
	Metastasis	0.114	<0.001
Platelet, 10^9^/L	Non-metastasis	0.069	0.001
	Metastasis	0.079	0.018
Monocyte, 10^9^/L	Non-metastasis	0.105	<0.001
	Metastasis	0.385	<0.001

UAR, Uric acid to Albumin Ratio; CEA, carcinoembryonic antigen; CA199, carbohydrate antigen 199; CA724, carbohydrate antigen 724; NLR, Neutrophil to Lymphocyte Ratio.

**Table 2:** Demographic and laboratory characteristics of patients.

Characteristics	Non-metastasis (n=332)	Metastasis (n=156)	*P*-value
Sex, no. (%)			0.556
Male	245(73.8)	119(76.3)	
Female	87(26.2)	37(23.7)	
Age, years	61(52-67)	59(51.3-68)	0.943
Albumin, g/L	37.6 ± 4.6	37.5 ± 4.7	0.748
Globulin, g/L	25.2 ± 4.1	26.2 ± 4.0	0.011
Uric acid, umol/L	263.3(215.2-315.1)	312.1(268.2-378.0)	<0.001
UAR	7.01(5.7-8.5)	8.58(6.9-10.1)	<0.001
CEA, ng/mL	1.68(3.4-59.7)	7.12(3.0-109.7)	<0.001
CA199, U/mL	12.88(6.2-207.2)	26.98(10.5-141.6)	0.006
CA724, U/mL	4.33(1.9-28.8)	10.1(2.4-34.9)	0.005
Lymphocyte, 10^9^/L	1.47(1.1-2.0)	0.72(0.4-1.6)	<0.001
Neutrophil, 10^9^/L	3.19(2.4-4.2)	3.54(2.8-4.7)	0.004
NLR	2.02(1.4-3.3)	4.23(2.2-8.5)	<0.001
Erythrocyte, 10^12^/L	4.24(3.8-4.7)	3.9(3.4-4.4)	<0.001
Hemoglobin, g/L	127(107.0-141.0)	133(104.3-155.0)	0.017
Leukocyte, 10^9^/L	5.44(4.3-6.8)	5.51(4.5-6.8)	0.488
Platelet, 10^9^/L	206(162.3-260.0)	195(140.3-251.5)	0.025
Monocyte, 10^9^/L	0.42(0.3-0.6)	0.45(0.3-0.6)	0.083

### UA, UAR and NLR can predict more effectively colorectal cancer bone metastases than tumor maker

**Figure 2:**
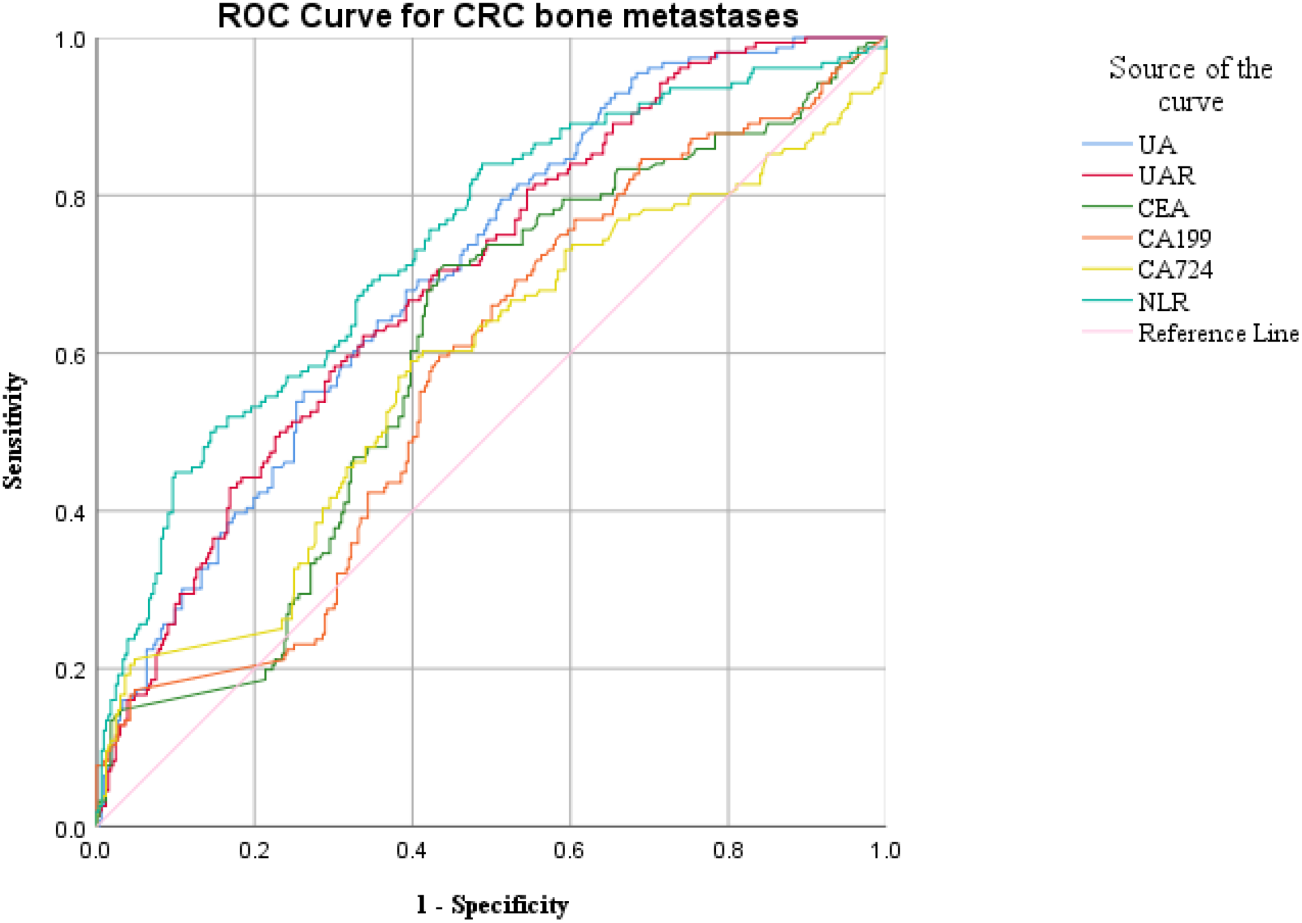
ROC analysis for the prediction of CRC bone metastasis. AUC indicates the diagnostic power of UA, UAR, NLR, CEA, CA199 and CA724 for bone metastasis.

ROC curve analysis was employed to assess the diagnostic efficacy of various parameters for detecting bone metastasis in CRC. The optimal cut-off values for UA, UAR, NLR, CEA, CA199 and CA724 were determined to be 309.9 (sensitivity 55.1%, specificity 74.1%), 7.69 (sensitivity 62.2%, specificity 66.5%), 4.201 (sensitivity 50.6%, specificity 84.9%), 3.97 (sensitivity 71.2%, specificity 56.2%), 9.975 (sensitivity 76.9%, specificity 39.3%) and 7.79 (sensitivity 56.0%, specificity 60.2%), respectively. The AUC values for UA, UAR, NLR, CEA, CA199 and CA724 in predicting bone metastasis were 0.705 [95% CI: 0.658–0.752, P < 0.001], 0.698 [95% CI: 0.650–0.746, P < 0.001], 0.738 [95% CI: 0.690–0.786, P < 0.001], 0.602 [95% CI: 0.549–0.655, P < 0.001], 0.576 [95% CI: 0.522–0.630, P = 0.007] and 0.578 [95% CI: 0.521–0.635, P = 0.005]. The predictive efficacy of UA, UAR and NLR was found to be comparable to that of established CRC tumor markers (**Figure 2**). The predicted probabilities for combining CRC markers with UA, UAR and NLR was derived using binary logistic regression. The optimal cut-off values for P1, P2, P3, P4 and P5 were identified as 0.278 (sensitivity 70.5%, specificity 54.3%), 0.248 (sensitivity 80.1%, specificity 51.0%), 0.301 (sensitivity 62.8%, specificity 70.5%), 0.330 (sensitivity 53.8%, specificity 87.1%) and 0.218 (sensitivity 75.0%, specificity 76.0%). The AUC values for P1, P2, P3, P4 and P5 were 0.628 (95% CI: 0.576–0.680, P < 0.001), 0.723 (95% CI: 0.676–0.770, P < 0.001), 0.719 (95% CI: 0.671–0.767, P < 0.001), 0.759 (95% CI: 0.712–0.807, P < 0.001) and 0.825 (95% CI: 0.787–0.863, P < 0.001) (**Table 3**). As indicated in **Table 4**, significant differences were observed except for three pairs P2 vs P3, P2 vs P4, P3 vs P4). The similar AUCs of P2 (AUC = 0.723), P3 (AUC = 0.719) and P4 (AUC = 0.759) suggested their equivalent diagnostic accuracy for bone metastasis in CRC. Notably, the combination of UA, UAR, NLR and tumor markers significantly enhanced diagnostic efficacy (**Figure ​3**; **Table 4**).

**Table 3:** The ROC curve determined the optimal cut-off values for the diagnostic parameters of bone metastases in colorectal cancer.

Parameters	AUC	95% CI	Cut-off	Sen	Spe	Youden index	P value
UA	0.705	0.658-0.752	309.9	0.551	0.741	0.291	<0.001
UAR	0.698	0.650-0.746	7.69	0.622	0.665	0.287	<0.001
CEA	0.602	0.549-0.655	3.97	0.712	0.562	0.273	<0.001
CA199	0.576	0.522-0.630	9.975	0.769	0.393	0.162	0.007
CA724	0.578	0.521-0.635	7.79	0.56	0.602	0.191	0.005
NLR	0.738	0.690-0.786	4.201	0.506	0.849	0.355	<0.001
P1	0.628	0.576-0.680	0.278	0.705	0.543	0.247	<0.001
P2	0.723	0.676-0.770	0.248	0.801	0.51	0.31	<0.001
P3	0.719	0.671-0.767	0.301	0.628	0.705	0.333	<0.001
P4	0.759	0.712-0.807	0.33	0.538	0.871	0.409	<0.001
P5	0.825	0.787-0.863	0.281	0.75	0.76	0.509	<0.001

AUC, Area under receiver operating characteristics; CI, Confidence Interval; UA, Uric acid; Sen, Sensitivity; Spe, Specificity; P1, Prediction probability obtained by binary logistic regression combining CEA, CA199 and CA724; P2, Prediction probability obtained by binary logistic regression combining CEA, CA199, CA724 and UA; P3, Prediction probability obtained by binary logistic regression combining CEA, CA199, CA724 and UAR; P4, Prediction probability obtained by binary logistic regression combining CEA, CA199, CA724 and NLR; P5, Prediction probability obtained by binary logistic regression combining CEA, CA199, CA724, UA, UAR and NLR.

**Table 4:** Comparison of AUC values between any two of the P1-P5.

	DBA (95% CI)	*P* value
P1 vs P2	-0.095 (-0.153, -0.037)	0.001
P1 vs P3	-0.091 (-0.148, -0.034)	0.002
P1 vs P4	-0.131 (-0.188, -0.075)	<0.001
P1 vs P5	-0.197 (-0.252, -0.142)	<0.001
P2 vs P3	0.004 (-0.017, 0.026)	0.693
P2 vs P4	-0.036 (-0.100, 0.027)	0.264
P2 vs P5	-0.102 (-0.141, -0.063)	0.001
P3 vs P4	-0.041 (-0.105, 0.024)	0.215
P3 vs P5	-0.106 (-0.144, -0.068)	<0.001
P4 vs P5	-0.065 (-0.102, -0.029)	<0.001

*DBA* Difference between AUCs, AUCs were compared via Delong test.

*CI* Confidence interval, *AUC* Area under the curve.

**Figure 3:**
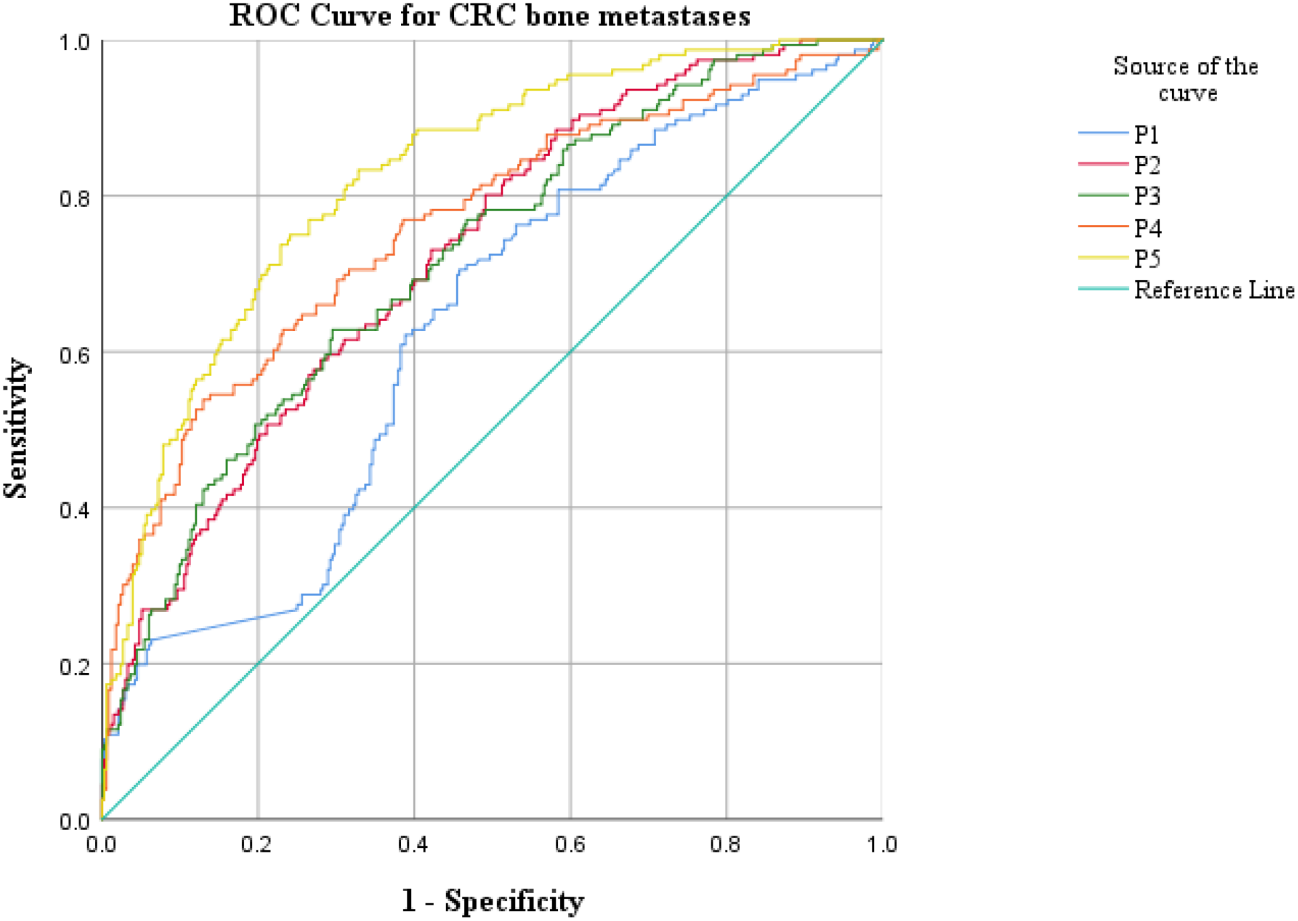
ROC analysis for the prediction of CRC bone metastasis. AUC indicates the diagnostic power of prediction probability for bone metastasis. P1: Prediction probability obtained by binary logistic regression combining CEA, CA199 and CA724; P2: Prediction probability obtained by binary logistic regression combining CEA, CA199, CA724 and UA; P3: Prediction probability obtained by binary logistic regression combining CEA, CA199, CA724 and UAR; P4: Prediction probability obtained by binary logistic regression combining CEA, CA199, CA724 and NLR; P5: Prediction probability obtained by binary logistic regression combining CEA, CA199, CA724, UA, UAR and NLR.

The correlation among UA, UAR, NLR and other diagnostic parameters were presented in **Table ​5**. In patients with CRC, UA and UAR exhibited positive correlations with CEA and CA724 (r = 0.091, 0.102, 0.106 and 0.124, respectively, all P < 0.05). However, no significant correlation was observed between UA and CA199 (P = 0.205). Additionally, NLR demonstrated positive correlations with CEA, CA199 and CA724 (r = 0.122, 0.093 and 0.136, respectively, all P < 0.05).

**Table 5:** Correlation between UA, UAR, NLR and diagnostic parameters of bone metastases in colorectal cancer.

	Spearman correlation		
	UA	UAR	NLR
UA		0.908^**^	0.066
UAR	0.908^**^		0.086
NLR	0.066	0.086	
CEA	0.091^*^	0.106^*^	0.122^**^
CA199	0.05	0.025	0.093^*^
CA724	0.102^*^	0.124^**^	0.136^**^

*/**Statistically significant. **P-value<0.01; *P-value<0.05.

### UA and NLR can be independent risk factors for bone metastasis of colorectal cancer

The diagnostic parameters were categorized based on their optimal cut-off values, resulting in two groups: high and low. Univariate logistic regression analysis revealed that elevated UA (OR: 3.46, 95% CI: 2.321-5.157, P < 0.001), elevated UAR (OR: 3.186, 95% CI: 2.146-4.731, P < 0.001), elevated NLR (OR: 5.786, 95% CI: 3.747-8.937, P < 0.001), elevated CEA (OR: 3.142, 95% CI: 2.088-4.728, P < 0.001), elevated CA199 (OR: 2.174, 95% CI: 1.41-3.348, P < 0.001) and elevated CA724 (OR: 2.178, 95% CI: 1.478-3.209, P< 0.001) were identified as risk factors for colorectal cancer metastasis (**Table 4**). Furthermore, multivariate logistic regression analysis indicated that elevated UA (OR: 2.73, 95%CI: 1.399-5.328, P = 0.003), elevated NLR (OR: 6.42, 95%CI: 3.914-10.532, P < 0.001) and elevated CEA (OR: 2.365, 95%CI: 1.423-3.93, P = 0.001) were independent risk factors for colorectal cancer bone metastasis (**Table 6**).

**Table 6:** Univariate and multivariate binary logistic regression analyses of variables for colorectal cancer bone metastases.

	Univariate analysis			Multivariate analysis		
	Odds ratio	95% CI	*P* value	Odds ratio	95% CI	*P* value
Age, years						
≥67.5 vs. <67.5	1.377	0.903-2.101	0.137	1.133	0.687-1.869	0.624
UA						
High vs. low	3.46	2.321-5.157	<0.001	2.73	1.399-5.328	0.003
UAR						
High vs. low	3.186	2.146-4.731	<0.001	1.836	0.944-6.57	0.074
NLR						
High vs. low	5.786	3.747-8.937	<0.001	6.42	3.914-10.532	<0.001
CEA						
High vs. low	3.142	2.088-4.728	<0.001	2.365	1.423-3.93	0.001
CA199						
High vs. low	2.172	1.41-3.348	<0.001	1.504	0.887-2.55	0.13
CA724						
High vs. low	2.178	1.478-3.209	<0.001	1.008	0.619-1.64	0.976

The reference of age, NLR, CEA, CA19–9 and CA72–4 was age < 67.5, UA < 309.9, UAR < 7.69, NLR < 4.201, CEA < 3.97, CA199 < 9.975 and CA724 < 7.79.

## Discussion

In our study, we investigated serum UA levels, UAR and NLR in patients diagnosed with colorectal cancer. Our finding indicated that serum UA, UAR, NLR and CEA levels were significantly elevated in patients with bone metastatic colorectal cancer compared to those without such metastasis. Furthermore, multiple logistic regression analysis revealed that higher serum UA, NLR and CEA levels were associated with bone metastatic colorectal cancer. However, we did not observe a significant association between UAR and tumor bone metastasis in this patient population.

Previous prospective studies have demonstrated that elevated serum UA level was associated with poorer prognoses in cancer patients (5). Additionally, serum UA level has been identified as an independent risk factor for esophageal carcinoma, colorectal cancer and oral squamous cell carcinoma (12–14). Currently, there was limited research on the relationship between serum UA levels and tumor bone metastasis in colorectal cancer patients. The findings of this study indicated that serum UA level in the bone metastasis group was significantly higher than that in the group without bone metastasis. Univariate logistic regression analysis revealed that serum UA levels exceeding 309.9 umol/L were linked to an increased likelihood of bone metastasis. Furthermore, multivariate logistic regression analysis suggested that individuals with elevated UA levels were more susceptible to a higher incidence of bone metastasis, with the difference reaching statistical significance. In summary, this study identified elevated UA levels as a risk factor for bone metastasis in colorectal cancer, suggesting that high UA levels may serve as a predictor for such metastasis. Previous research indicated that elevated uric acid concentrations can induce inflammation and oxidative stress, promote tumor cell proliferation and angiogenesis, and facilitate the invasion and metastasis of tumor cells (15, 16). However, there is a paucity of studies examining the relationship between serum uric acid and bone metastasis, and the underlying molecular mechanisms remain unclear. Further, basic research and large-scale cohort studies are necessary to investigate.

Hypoalbuminemia represents another adverse prognostic factor for colorectal cancer. In survival studies involving patients with colorectal cancer who received both surgical and non-surgical treatments, albumin has been utilized either as a component of the study or as part of a prognostic score. Li X et al. demonstrated that a high albumin-to-globulin ratio serves as a reliable indicator of overall survival and disease-free survival (17). Our research indicated that, while low albumin levels alone did not significantly predict bone metastases in colorectal cancer, the UAR was significantly associated with such metastases, as revealed by further ROC analysis. In our study, the incidence of bone metastases was notably higher in the high UAR group. To date, no studies have reported a correlation between UAR and bone metastases in colorectal cancer. To our knowledge, this investigation was the first to establish that elevated UAR levels predict bone metastases in colorectal cancer.

Studies have demonstrated a close relationship between inflammation and tumors (18). Previous research has indicated that the proportion of neutrophils in peripheral blood increases as malignant tumors progress (19, 20). An elevated NLR signifies either a relative increase in neutrophils numbers or a relative decrease in lymphocytes counts. Evidence suggests that neutrophils can enhance the release of inflammatory mediators and facilitate tumor neovascularization. Conversely, neutrophils may diminish the body’s anti-tumor capacity by inhibiting the activity of lymphocytes and natural killer cells, thereby promoting distant tumor metastasis. Lymphocytes not only inhibit tumor cell proliferation and metastasis, but also directly induce tumor cells death (21). A decrease in lymphocyte numbers indicated a weakened anti-tumor immune function, which can lead to tumor invasion and progression. The finding of this study revealed that the NLR in the bone metastasis group was significantly higher than that in the non-bone metastasis group, establishing a close association between NLR and bone metastasis in colorectal cancer. Consequently, a high NLR is more likely to be associated with bone metastasis, suggesting that NLR may serve as a novel marker for assessing tumor bone metastasis in patients with colorectal cancer.

The predictive and prognostic effects of UA and NLR in cancer have been extensively investigated. Most studies have concentrated on elevated UA and NLR as indicators of long-term survival in cancer patients or have examined the role of NLR in assessing lymph node metastasis (10, 22–24). However, the predictive capacity of UA and NLR in evaluating bone metastasis has received comparatively less attention. While the involvement of UA and NLR in distant metastases among tumor patients has been documented, researchers have not yet compared the diagnostic efficacy of UA and NLR with traditional tumor markers. In our study, we demonstrated that UA and NLR outperformed conventional tumor markers in assessing bone metastasis. The optimal cut-off values for UA and NLR were determined to be 309.9 umol/L and 4.201, respectively, with sensitivities of 55.1% and 50.6%, and specificities of 74.1% and 84.9%. Among these indices, the combination of UA, the UAR, NLR and tumor markers exhibited the highest predictive accuracy for bone metastasis in CRC, whereas CA199 and CA724 demonstrated the lowest performance as indicated by logistic regression and ROC analysis. Further research is requires to ascertain whether the detection and targeted therapy of UA and neutrophils can enhance the prognosis of colorectal cancer and facilitate clinical treatment.

However, our study has several limitations. First, it is a single-center retrospective analysis, which may introduce bias and errors. The conclusions further validation through additional basic studies, as well as multi-center and large-sample cohort studies. Second, certain confounders related to UA, such as diet, exercise, and alcohol consumption, were not included in the analysis. Furthermore, additional clinical parameters reflecting disease severity are necessary to elucidate the relationships among UA, UAR NLR and bone metastatic status in the multivariate regression analysis. Finally, we analyzed the relationships among UA, UAR, NLR and clinical prognosis in CRC patients. Despite these limitations, our findings indicate that UA, UAR and NLR may serve as novel markers for assessing tumor bone metastasis in patients with CRC.

## Conclusion

In summary, our findings indicate that the combination of UA, UAR, NLR and tumor markers exhibits the highest diagnostic performance for bone metastasis in CRC. Both UA and NLR serve as valuable indicators for predicting bone metastases in CRC patients. Consequently, clinicians should closely monitor patients with UA levels exceeding 309.9 umol/L and NLR values greater than 2.91. Furthermore, they should undertake additional examinations to identify bone metastases at the earliest opportunity.

## Data Availability

The original contributions presented in the study are included in the article/supplementary material. Further inquiries can be directed to the corresponding author.
